# In Silico Study Suggesting the Bias of Primers Choice in the Molecular Identification of Fungal Aerosols

**DOI:** 10.3390/jof7020099

**Published:** 2021-01-30

**Authors:** Hamza Mbareche, Marc Veillette, Guillaume J. Bilodeau

**Affiliations:** 1 Sunnybrook Research Institute, Toronto, ON M4N 3M5, Canada; 2 Department of Laboratory Medicine and Pathobiology, University of Toronto, Toronto, ON M5S, Canada; 3 Centre de Recherche de L’institut Universitaire de Cardiologie et de Pneumologie de Québec, Quebec City, QC G1V 4G5, Canada; Marc.Veillette@criucpq.ulaval.ca; 4 Pathogen Identification Research Lab, Canadian Food Inspection Agency (CFIA), Ottawa, ON K2H 8P9, Canada; guillaume.bilodeau@canada.ca

**Keywords:** bioaerosols, fungi, ITS1, ITS2, UNITE, database

## Abstract

This paper presents an in silico analysis to assess the current state of the fungal UNITE database in terms of the two eukaryote nuclear ribosomal regions, Internal Transcribed Spacers 1 and 2 (ITS1 and ITS2), used in describing fungal diversity. Microbial diversity is often evaluated with amplicon-based high-throughput sequencing approaches, which is a target enrichment method that relies on the amplification of a specific target using particular primers before sequencing. Thus, the results are highly dependent on the quality of the primers used for amplification. The goal of this study is to validate if the mismatches of the primers on the binding sites of the targeted taxa could explain the differences observed when using either ITS1 or ITS2 in describing airborne fungal diversity. Hence, the choice of the pairs of primers for each barcode concur with a study comparing the performance of ITS1 and ITS2 in three occupational environments. The sequence length varied between the amplicons retrieved from the UNITE database using the pair of primers targeting ITS1 and ITS2. However, the database contains an equal number of unidentified taxa from ITS1 and ITS2 regions in the six taxonomic levels employed (phylum, class, order, family, genus, species). The chosen ITS primers showed differences in their ability to amplify fungal sequences from the UNITE database. Eleven taxa consisting of Trichocomaceae, Dothioraceae, Botryosphaeriaceae, Mucorales, Saccharomycetes, Pucciniomycetes, Ophiocordyceps, Microsporidia, Archaeorhizomycetes, Mycenaceae, and Tulasnellaceae showed large variations between the two regions. Note that members of the latter taxa are not all typical fungi found in the air. As no universal method is currently available to cover all the fungal kingdom, continuous work in designing primers, and particularly combining multiple primers targeting the ITS region is the best way to compensate for the biases of each one to get a larger view of the fungal diversity.

## 1. Introduction

Bioaerosols are airborne biological particles found almost everywhere, and they are influenced by the surrounding sources [1]. These particles are composed of a mixture of dead and living organisms (e.g., viruses, bacteria, fungi) and their components (e.g., toxins, proteins, metabolites) [2]. Fungi are one of the most diverse group of living organisms on the planet [3], and it could be reflected in the air as well [4]. Traditionally, researchers relied on the isolation of fungal species using culture based methods. However, only culturable fungal species collected from air samples to study their concentration and diversity induce a bias. The gap in the literature induced by the application of cultivation methods solely is linked to the specific representation of the cultivable and viable portion of fungal aerosols. Fabian and collaborators showed that the percentage of culturability for biological structures in outdoor air was 0.1% for bacteria (on trypticase soy agar medium), and 0.001% for fungi (on malt extract agar medium) [5]. The underrepresented portion of the fungal community might have a significant role in the adverse health effects related to exposure, especially that some negative health impacts, like allergies, do not rely exclusively on the viability of microbes. Thus, it is necessary to consider not only the tip of the iceberg, but look at the bigger picture of fungal aerosols.

The enthusiasm to fill the gap of bioaerosol studies with the nonviable or the uncultivable portion of fungal turned the attention to the high-throughput sequencing (HTS) of the genomic DNA extracted from aerosol samples, as streamlined by targeting fungal universal barcodes [6,7]. A recent study compared the performance of the Internal Transcribed Spacers 1 and 2 (ITS1 and ITS2), the two universally used barcodes to describe fungal diversity latterly, in the air of three different occupational environments [8]. The main conclusion was that although ITS1 performed better by giving richer and more diverse fungal air samples, both barcodes gave different taxonomic profiles. Therefore, using ITS1 and ITS2 offered a broader view of the identified taxa. This conclusion is supported by previous in silico studies [9]. Currently, the fungal ecology community largely uses ITS2 for metabarcoding because the ITS1 subregion is more variable in length, and longer amplicons are not advantaged in PCR steps, which leads to taxonomic biases against, e.g., Boletales. Few studies have highlighted that the primers for ITS2 are more general and less prone to taxonomic bias than are those for ITS1 [10,11].

The difference in taxonomic profiles obtained by both barcodes could be associated with the representativeness of the barcodes in the database. The most used database for molecular identification of fungi is UNITE (https://unite.ut.ee/), a web-based and sequence management environment for ITS region [12]. Another hypothesis is that the primer choice targeting ITS1 or ITS2 in the amplification step prior to sequencing could be the cause of the differences observed between the taxonomic profiles obtained by both barcodes. The aim of this study (2) was to evaluate (1) the current state of the latest version of the UNITE database in terms of representative sequences of ITS1 and ITS2, (2) if the mismatches of the primers in the binding sites of the targeted taxa could explain the differences between the two barcodes applying an in silico analysis. To be more concise, we only used the pairs of primers that were applied in the study comparing the performance of ITS1 and ITS2 in three occupational environments [8]. The choice of the primers in the latter study was based on a large-scale study by Tedersoo et al. comparing an extended set of ITS primers [13]. We evaluated also the length of the targeted sequences of ITS1 and ITS2 in the database, as shorter DNA fragments are preferentially amplified in complex mixtures like environmental samples [14]. It is important to note that this study does not intend to present the biases introduced by the broad ITS primers selection. Previous studies by Tedersoo et al. 2015, and Nilsson et al. 2019 produced a list of different pairs of primers and reported potential biases in taxa identification [13,15]. The work presented herein is an extension of the original results of airborne fungi presented in Mbareche et al., 2020 and shed light on possible reasons behind the difference between ITS1 and ITS2 in studying fungal diversity. In addition, the actual work adds another piece to the puzzle comparing ITS regions in fungi identification, particularly in aerosols. 

## 2. Methods 

### 2.1. UNITE Dataset

At the time of the analysis, the latest UNITE database was released on 2 February, 2019. Thus, the UNITE version included in this study is ver8_02.02.2019. Because most of the diversity analyses in the study comparing the performance of ITS1 and ITS2 in the three occupational environments (compost, biomethanization and dairy farms) [8] was done using QIIME 1.9.1, the UNITE version released by QIIME (sh_refs_qiime_ver8_97_02.02.2019) was used in this study as well for consistency. The in silico analysis requires the use of a dataset containing the binding sites of the primers (parts of SSU 18S and LSU 28S). Therefore, the untrimmed version of the QIIME released UNITE database was used. Usually, all sequences were run through ITSx 1.1.2 [16] to remove larger parts of the 18S and the 28S as they may affect clustering and sequence identification. Considering the aim of this study, conserving the primer binding sites was mandatory for the success of the analyses. In addition, the present study used reference sequences after 97% similarity clustering (sequences that 97% similar formed the same cluster). No single similarity threshold can accurately reflect the species level throughout the whole fungal kingdom, but the UNITE version was chosen where the species are identified with the same threshold as the one applied for OTU clustering in most of our studies (97%). The raw data used in this study are publicly available at https://unite.ut.ee/repository.php. 

### 2.2. In Silico Amplification 

The ecoPCR 1.01 software developed by the Laboratoire d’Écologie Alpine, Grenoble, France (Ficetola et al., 2010) was used for the in silico amplification of the ITS sequences from the UNITE database using two pairs of primers targeting ITS1 and ITS2 (Table 1) [17]. The download, the installation and the documentation of ecoPCR can be found at the GitLab page: https://git.metabarcoding.org/obitools/ecopcr/wikis/home. The ecoPCR package contains different command line-based tools. ecoPCRFormat compiles the UNITE database into the acceptable format of ecoPCR using the default parameters. In brief, ecoPCR selects sequences from a database that exhibit similarity to two PCR primers. With OBITools, the user can compute barcode coverage and barcode specificity [17]. The required inputs are the formatted database to test the primers against and the primer sequences. Different options allow determination of minimum and maximum amplicon length, the number of mismatches and their positions, and restrictions to specific taxonomic groups that the user want to exclude from the analyses. The ecoPCR output contains the amplification length, the melting temperature (Tm), the taxonomic identification, and the number of mismatched positions for each sequence. Since Tm is affected by the strength of hybridization between the primers and the DNA template, it is important to show how the Tm decreases with the increase of the number of mismatches between the primers and the targeted sequences. This informs on the stringency of the PCR conditions. For each simulated amplification, up to three mismatches were allowed between each primer and the binding site of the targeted sequences on the database to emulate environmental conditions and limit the biases of PCR conditions that are too stringent. Two conditions were added: (1) excluding three successive mismatches, (2) excluding mismatches in the two bases of the 3′ primer end.

Statistical analysis was done using an R script with R version 3.3.3 and RStudio version 1.0.153. 

### 2.3. Fungal Bioaerosol Data

The primers used in the actual in silico analysis are the same ones used in Mbareche et al., 2020 [8], comparing ITS1 and ITS2 for the description of fungal diversity in bioaerosols collected from three different environments. In total, 50, 32, and 16 air samples were collected from compost, biomethanization and dairy farms, respectively. Bioaerosols were collected using a liquid cyclonic impactor Coriolis µ^®^ (Bertin Technologies, Montigny-le-Bretonneux, France). The sampler was set at 200 L/min for 10 min (2m^3^ of air per sample) and placed within 1–2 m of the bioaerosol source. The sampling sites were chosen according to workers’ activities. DNA extraction and HTS methods are detailed in the publication [8]. Data is available at National Center for Biotechnology Information (NCBI) under the BioProject ID: PRJNA533145.

## 3. Results

### 3.1. Amplicon Length

The sequence length varied between the amplicons retrieved from the UNITE database using the pair of primers targeting ITS1 and ITS2. When all taxonomic groups were considered, ITS2 fragments were longer than ITS1 (*p* = 0.04, two tailed T-test). In the most abundant phyla (Ascomycota and Basidiomycota), ITS1 fragments had similar length across all the taxa (*p* = 0.5, two-tailed T-test) but, ITS2 fragments showed differences between the two phyla, with longer fragments in Basidiomycota compared to Ascomycota (*p* = 0.01, two-tailed T-test). 

### 3.2. Representative Sequences of ITS1 and ITS2 in UNITE

**Figure 1:**
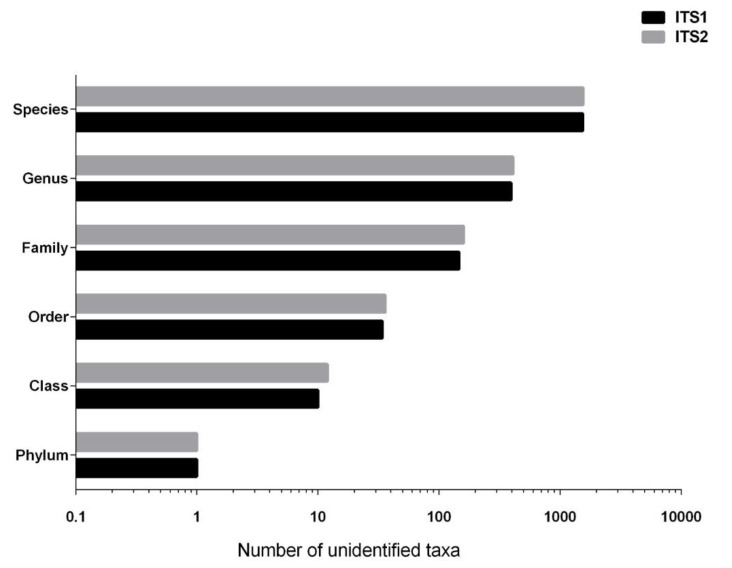
Number of unidentified taxa (sequences that could not be identified or that lack full species name) in all taxonomic level of the UNITE database. Data was separated according to the sequences retrieved with primers targeting ITS1 and ITS2.

**Figure 2:**
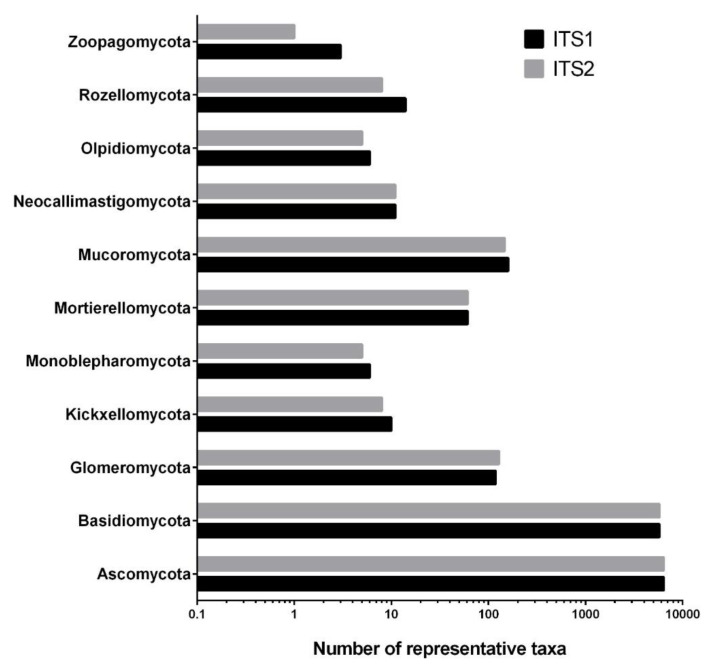
Number of representative taxa in the 11 phyla of the UNITE database. Data was separated according to the sequences retrieved with primers targeting ITS1 and ITS2.

First, the number of unidentified taxa (sequences that could not be identified or that lack full species name) retrieved after using the pairs of primers targeting ITS1 and ITS2 on the UNITE database was looked at. As can be seen in Figure 1, the results demonstrated that the database contains an equal number of unidentified taxa from ITS1 and ITS2 regions in the six taxonomic levels (phylum, class, order, family, genus, species). In some cases, the number of unidentified taxa was higher with ITS2 (two more unidentified classes, 13 more unidentified families, and 16 more unidentified species compared to ITS1). Second, we assessed the number of representative taxa from ITS1 and ITS2 region in each one of the 11 phyla present in UNITE (Figure 2). Again, ITS1 and ITS2 had a fairly comparable number of sequences identified in UNITE. However, some differences were noted, like Glomeromycota having 128 sequences in ITS2 compared to 119 in ITS1, and Rozellomycota having 14 sequences in ITS1 compared to eight sequences in ITS2. Further, Mucoromycota had 160 sequences in ITS1 compared to 147 in ITS2. Another notable difference is Zoopagomycota having three representatives in ITS1 and only one identified sequence in ITS2. 

### 3.3. Taxonomic Bias of Primer Choice 

**Figure 3:**
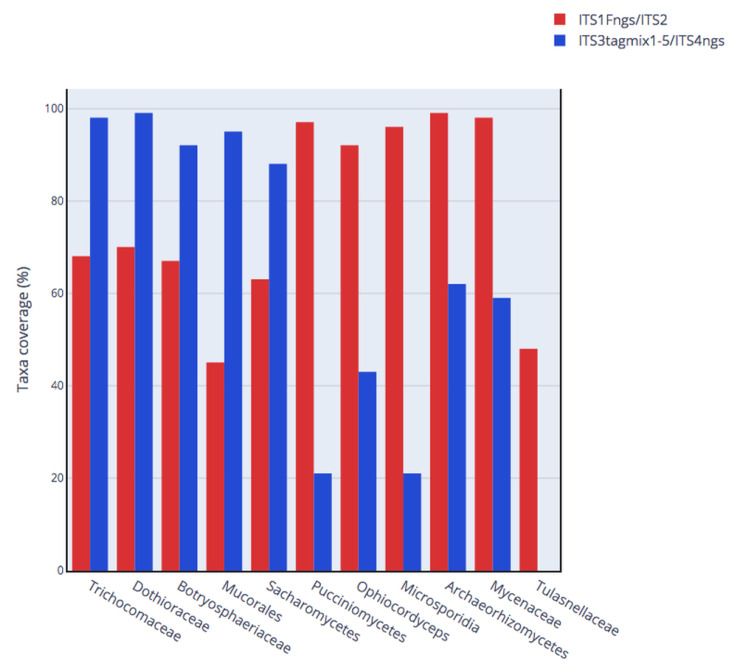
Percentage of sequences amplified (fungal taxa covered) from the UNITE database using two different pairs of primers targeting ITS1 (red) and ITS2 (blue). A maximum of three mismatches was allowed for the in silico amplification.

The chosen ITS primers in this study showed differences in their ability to amplify fungal sequences from The UNITE database. Figure 3 presents the percentage of fungal taxa covered by the pair of primers targeting ITS1 or ITS2. We focused on the 11 taxa that showed large variations between the two regions. The pair of primers targeting ITS2 amplified above 90% of Trichocomaceae, Dothioraceae, Botryosphaeriaceae, Mucorales, and Sacharomycetes. These same fungal taxa were amplified at 43% to 70% by the primers targeting ITS1. Contrarily, the pair of primers targeting ITS1 amplified above 90% of Pucciniomycetes, Ophiocordyceps, Microsporidia, Archaeorhizomycetes, and Mycenaceae. Whilst the primers targeting ITS2 amplified between 20% to 59% of the latter cited fungal taxa. Another striking example of the difference between the two regions is Tulasnellaceae, which was not amplified by the ITS2 primers (0%), while ITS1 primers amplified 48% of the sequences. Tulasnella and Epulorhiza have deviant 3′ portions of the 5.8S gene—this might explain why ITS2 is hard to amplify in Tulasnellaceae [18].

In the published work comparing the performance of ITS1 and ITS2 in three occupational environments (compost, biomethanization and dairy farms) one of the noted taxonomic variations between the two regions is that *Aspergillus* sp. were biased towards ITS1 and *Penicillium* sp. were biased towards ITS2 (Mbareche et al., 2020). Interestingly, the same primers were used in this work on the UNITE database and primers targeting ITS1 amplified 89% of *Aspergillus* and 68% of *Penicillium*. Conversely, primers targeting ITS2 amplified 92% of *Penicillium* sequences in UNITE and 56% of *Aspergillus* sequences.

## 4. Discussion

The ITS region has been widely used to explore fungal diversity, especially since 2012 when a study showed its usefulness for describing fungi compared to other genetic markers [19]. Airborne fungi were essentially studied using culture methods, until the burst of HTS in recent years. Amplicon-based HTS methods targeting the ITS region focuses on ITS1 or ITS2 to describe fungal diversity because the sequencing platforms widely used have limitations in the amplicon length, and cannot cover the whole ITS region. The difference noted between ITS1 and ITS2 for exploring fungal diversity in aerosols could be related to the fungal databases and the amplification primers. This study proved that the choice of primers is responsible for the taxonomic variation when we use ITS1 or ITS2, essentially because of primer mismatch bias towards specific fungal groups.

The noted longer length of ITS2 sequences from air samples in three different environments compared to ITS1 [8] was confirmed in this study, as ITS2 sequences in the UNITE database were also longer than ITS1. The length difference could be one of the reasons why ITS1 (being shorter and preferentially amplified) performed better at covering the diversity of air samples than ITS2. However, the difference in the taxonomic identification could not be explained by the number of unidentified taxa nor by the sequence representation of the two regions in the UNITE database, because both regions had roughly equal representations of identified and unidentified sequences. It is noteworthy that the phyla Rozellomycota, Zoopagomycota, Olpidomycota and Neocallimastigomycota have an underrepresented number of sequences in the UNITE database. This addresses the question if the UNITE database is updated with new taxonomic information in all phyla and not only the most studied ones (Ascomycota and Basidiomycota). This underrepresentation could also be that the latter phyla are simply understudied, hence undersequenced. 

The in silico analysis of this study is a confirmation and a continuity of previous results about ITS primers biases by Bellemain et al., 2010, Op De Beeck et al., 2014, and Tedersoo et al., 2015 [10,13,20]. It extends the results of previous studies by adding additional fungal taxa, more commonly found in the air, affected by the choice of primers like ITS1 being biased towards *Aspergillus* and ITS2 towards *Penicillium* and Saccharomycetes. Also, most of the previous studies comparing the two barcodes focused on soil, mangroves, plants, or aquatic ecosystems. Because many factors can affect fungal recovery from air samples for molecular biology analyses (e.g., air sampling devices, sample processing, DNA extraction), it is important to address the bias in the choice of primers affecting fungal taxa recovered from air samples particularly. The fact that the in silico results coincide with the results obtained in environmental air samples confirmed our hypothesis. In this study, we used the UNITE database where all the sequences corresponding to species hypotheses (SHs) had a 97% sequence similarity threshold, a value that is popular in HTS studies. Because the 97% threshold is considered too low to separate closely related species, we considered only the fungal taxa at a lower taxonomic level (phylum, class, family, and genus). To overcome this issue, taxonomic experts should go through the taxonomic identification of fungi in UNITE and determine what threshold value should be used for each taxonomic group to delimit the difference between species. Thus, some SHs could be better delimited at 97%, others at 99% or even 100%. Such implementation could have strong benefits for future in silico studies aiming to evaluate and design better ITS primers, with the consideration of the species level. Kõljalg and colleagues implemented this work in 2013, which could be improved by expert in different fungal lineages through the web-based sequence management system in UNITE [21]. The results of the amplification against the UNITE database were affected by the number mismatches allowed between the pair of primers and the sequences in the database. Obviously, when zero mismatches were allowed the percentage of taxa covered (Figure 3) by the two pair of primers was low. For primers targeting both regions, the proportion of amplified sequences increased with the number of mismatches allowed. It should be mentioned that, as the percentage of taxa covered increased with the number of mismatches allowed, the relative difference between ITS1 and ITS2 for the specified fungal groups remained the same. Our results strongly suggest that it is important to use these primers under PCR conditions that are not too stringent to avoid losing a high percentage of fungal taxa in environmental samples. Finding the right balance of stringency is key to allow the right amount of targeted sequences to be amplified and avoiding unspecific amplification. Thus, amplicon-based HTS studies should include tests that will help find the right balance of stringency by allowing the right amount of targeted sequences to be amplified and avoiding unspecific amplification. These PCR conditions are directly affected by the Tm of the primers, as the annealing temperature (Ta) close or above the recommended Tm will not allow sequences including one or more mismatches. In general, it is recommended to use an annealing temperature about 5 °C below the Tm of the primers [22]. A high Tm will most likely hamper the overall fungal diversity of environmental samples. As was expected, our in silico analysis showed that Tm decreases with the increase of the number of mismatches between the primers and the targeted sequences. 

Many studies have addressed the comparison between ITS1 and ITS2 over the years in silico or in different environmental samples [23,24,25,26]. However, the literature on airborne fungi is scarce, and the rapid evolution of sequencing and its affordability calls for frequent updates on primer biases and databases, especially in amplicon-based HTS analyses of fungi. This work emphasizes the importance to use bioinformatics to select the best pair of primers in amplicon-based HTS. The results presented herein were deliberately limited to the pairs of primers applied to our analyses on the performance of ITS1 and ITS2 in describing fungal aerosols in three different environments, to validate the hypothesis made about the role of the primers in explaining the taxonomic variations obtained by the two barcodes. Thus, future work can be extended by including more primers targeting ITS1 and ITS2 to find which pairs complement each other by covering most taxonomic groups. In addition, the bias related to the imperfection of the UNITE database in terms of uncertainty or wrong identification is also involved in the accuracy of the in silico analysis. Also, the in silico analysis does not include PCR parameters that cannot be simulated and that affect the performance of the primers in vitro. Examples of such parameters include, salt and primer concentration and amplification programs. Thus, in silico followed by in vitro analyses are recommended to confirm the right choice of primers and limit the biases associated with the primers choice in amplicon-based HTS. 

## 5. Conclusions

The aims of the study were achieved as, first, we determined that ITS1 and ITS2 were equally represented in the UNITE database in terms of identified and unidentified taxa, and second, the mismatches of the primers in the binding sites of the targeted taxa could explain the differences between the two barcodes applying an in silico analysis. Thus, the difference in the taxonomic profiles is because of the primers choice rather than the representation of ITS1 and ITS2 sequences in The UNITE database. Also, ITS2 had longer sequences in silico (this study) and in vitro [8]. This could explain the advantage of ITS1 in amplicon-based HTS studies. As no universal method is currently available to cover all the fungal kingdom, continuous work in designing primers, and particularly combining multiple primers targeting the ITS region is the best way to compensate for the biases of each one to get a broader view of the fungal diversity. Lastly, this work was intended to be a confirmation of the hypothesis stated in previous analyses regarding the reasons of the different taxonomic profiles obtained when we target ITS1 or ITS2. As this goal was accomplished successfully, it opens the door to the importance of bioinformatic analyses to (1) select the right primers, (2) consider the right PCR conditions, and (3) to assess the biases linked to the primers choice in amplicon-based HTS studies.

## Figures and Tables

**Table 1:** Primers used for amplification of the Internal Transcribed Spacers 1 and 2 (ITS1 and ITS2) regions.

Primers Name	Features	Sequence	Barcode
**ITS1Fngs**	Fwd	ACACTCTTTCCCTACACGACGCTCTTCCGATCTGGTCATTTAGAGGAAGTAA	ITS1
**ITS2**	Rev	GTGACTGGAGTTCAGACGTGTGCTCTTCCGATCTGCTGCGTTCTTCATCGATGC	ITS1
**ITS3tagmix1-5**	Fwd	ACACTCTTTCCCTACACGACGCTCTTCCGATCTTAGACTCGTCATCGATGAAGAACGCAG	ITS2
**ITS4ngs**	Rev	GTGACTGGAGTTCAGACGTGTGCTCTTCCGATCTTTCCTSCGCTTATTGATATGC	ITS2
